# Discover high-risk factor combinations using Bayesian network from cohort data of National Stoke Screening in China

**DOI:** 10.1186/s12911-019-0753-8

**Published:** 2019-04-09

**Authors:** Xuemeng Li, Jianfei Pang, Mei Li, Dongsheng Zhao

**Affiliations:** 1 Information Center, Academy of Military Medical Sciences, Beijing, China; 2 China Stroke Data Center, Beijing, China

**Keywords:** National Stroke Screening, Bayesian network, High-risk factors

## Abstract

**Background:**

In recent years, the increasing incidence and prevalence of stroke has brought a heavy economic burden on families and society in China. The Ministry of Health of the Peoples’ Republic of China initiated the national stroke screening and intervention program in 2011 for stroke prevention and control. In the screening, only those who have been classified to “potential high-risk” group in preliminary screening need further examination and physician confirmation to determine the risk level of stroke in rescreening. However, at the beginning of the program, the “potential high-risk” classification method in the preliminary screening are determined by experts based on their experience. The primary aim of this study is to study the causality of stroke and risk factors in middle-aged population using the cohort data, and to explore whether the stroke screening and intervention program should include more precise “potential high-risk” evaluation criteria for this age group in preliminary screening.

**Method:**

We use the cohort data of screening between 2013 and 2017 in this study. After data cleaning, the cohort consists of 48,007 people aged from 40 to 59 who are free of stroke at baseline. We use Bayesian networks to develop models.

**Result:**

The results show that the stroke incidence in middle-aged population with certain two risk factors is higher than some of that with three factors, which is in keeping with our previous study results. We can take the ratio of the stroke incidence with combinations of risk factors and incidence without any of the risk factors as a variable threshold. By adjusting the threshold, we can get precise stroke preliminary screening criteria to achieve a balance between economy and efficiency.

**Conclusion:**

We find that the criteria used in preliminary screening are not reasonable enough. There is a need for national stroke screening and intervention program to further include some more important risk factors or combinations of two risk factors as classification criteria in the preliminary screening. The results of the study can directly guide stroke screening program in China to make the screening more accurate and efficient.

## Background

In recent years, China has witnessed an increase in the incidence of chronic diseases led by the economic changes, lifestyle shift and aging population. On the whole, about 13 million residents suffer from stroke in China [1]. Although the mortality of stroke has decreased, the incidence and recurrence rate of stroke remain high in China [2, 3]. According to the World Bank data, at least 50% of the chronic disease occurs in the labor force population (aged 15 to 64) at present. Reducing the incidence of stroke is a guarantee of quality of life for middle-aged people, which is important both for families and the society.

Experience in developed countries shows that stroke is preventable and controllable. The risk factor management has been proved to be crucial for the management of stroke. In order to investigate the prevalence, morbidity, recurrence rate, mortality and disability rate of stroke in Chinese, and to improve residents’ awareness, the Ministry of Health of the Peoples’ Republic of China initiated the national stroke screening and intervention program in 2011, supported by a nationwide stroke screening platform called CSDC (China Stroke Data Center) [4]. The integrated treatment units established by the program in hospitals, called stroke centers, are responsible for diagnosing possible risk factors. The stroke screening and intervention program now consists of more than 3000 hospitals in China, and has achieved remarkable results in the prevention and treatment of stroke. With the promotion of the program, in 2017, the number of intravenous thrombolysis for acute ischemic stroke in Tianjin alone reached 3940. In the stroke screening, the program creatively proposed a two-step screening method. In the preliminary screening, there are eight high risk factors, including hypertension, diabetes, atrial fibrillation, dyslipidemia, smoking, apparently overweight or obese, lack of exercise and positive family history of stroke. The definition of risk factors is described in Table 1. A person is considered “potential high-risk” if suffering from three or more than three of the risk factors or having history of stroke or transient ischemic attack (TIA) in preliminary screening. For those who have been classified to “potential high-risk” group, further examination (such as computed head tomography and MRI scans) and physician confirmation are needed to determine the risk level of stroke in rescreening. People suffering from two of the risk factors are considered “potential medium-risk”. People identified as “high-risk” in the rescreening are followed through telephone every 6 months, and the tests for their blood pressure, blood sugar, and blood lipid are performed every 12 months to make an intervention. People identified as “potential medium-risk” are seen for follow-up visits once every 12 months, and their control on blood pressure, blood sugar, and atrial fibrillation are intervened and guided. Compared with the economic burden brought by stroke (including direct and indirect economic burden), expense of rescreening (about 600 yuan per person) is significantly lower, and reasonable intervention for population identified as “medium-risk” and “high-risk” can effectively reduce the economic burden on families and the society. However, at the beginning of the stroke screening and intervention program, due to the lack of national stroke data as a support, the classification method was determined by experts based on their experience. Although these indicators take into account the simplicity of screening, they may bring about some inaccuracies. The risk factors for stroke are age-related. Considering that Chinese stroke patients are getting younger and the important role middle-aged people played in society and families, we need to further determine the indicators for them in preliminary screening to control their incidence of stroke and to ensure their quality of life.

**Table 1:** The definition of risk factors

Risk factor	Definition	Values
Hypertension	Blood pressure value≥140/90 mmHg and systolic pressure ≥ 140 mmHg and/or diastolic pressure ≥ 90 mmHg without taking anti-hypertensive drugs; with a history of hypertension or takes anti-hypertensive drugs within 2 weeks (blood pressure is taken after tranquillization three times in different days).	Yes/No
Atrial Fibrillation	Rapidly disordered fibrillation waves and disappearance of regular atrial electrical activity. (Those with a history of paroxysmal atrial fibrillation should be proved by the diagnosis of hospitals at county level or above.)	Yes/No
Smoking	Continuous or cumulative smoking for 6 months, smoking within 30 days prior to the survey, and smoking at least 1 cigarettes a day during the survey.	Yes/No
Dyslipidemia	Glycerin trilaurate TG ≥ 2.26 mmol/L; total cholesterol TCHO≥6.22 mmol/L; low density lipoprotein cholesterol LDL- C ≥ 4.14 mmol/L; high density lipoprotein cholesterol HDL-C < 1.04 mmol/L.	Yes/No
Diabetes	Fasting blood-glucose≥7.0 mmol/L; OGTT: 2 h blood-glucose ≥11.1 mmol/L; HbA1c > 6.5%; RBS ≥ 11.1 mmol/L for those with significant hyperglycemia or acute hyperglycemia symptoms; patients who have been diagnosed with diabetes and are being treated are considered to suffer from diabetes.	Yes/No
Lack of exercise	Taking exercise less than 3 times a week and less than 30 min each time for at least 1 year (those regularly participate in industrial and agricultural activities are excluded).	Yes/No
Obvious overweight or obesity	BMI(Body Mass Index) ≥ 26	Yes/No
Positive family history of stroke	Those whose parents, children, siblings (with same parents) have a history of stroke.	Yes/No

In recent years, the risk factors of stroke have been studied in various ways. The internationally accepted stroke risk scale includes the Framingham Stroke Scale, Pool Cohort Equations, Stroke Riskometer, CHADS2 Scale, and CHA2DS2-VASc Scale. The Framingham Stroke Scale [5] is originally proposed by Framingham and later modified by D’Agostino et al. [6], which is currently the most widely used stroke risk assessment tool in foreign countries. In China, Huang et al. [7] proves that the risk of stroke in Chinese is obviously overestimated using the improved Framingham Stroke Scale. The Pooled Cohort Equations (https://clincalc.com/cardiology/ascvd/pooledcohort.aspx) uses an online calculator or mobile phone software to assess the risk of atherosclerotic cardiovascular disease (ASCVD) in the next 10 years (fatal and non-fatal cardiovascular). But there have been some controversies since its publication, and some external populations have shown that this risk assessment model may overestimate the risk of atherosclerotic cardiovascular disease. The Stroke Riskometer (https://www.strokeriskometer.com/) [8] is proposed by scholars from the Auckland University of Technology in New Zealand in 2014. The model validation population contains 9501 healthy people (from New Zealand, Russia, and the Netherlands). But due to the lack of support from Chinese data, the model still needs to be improved. The CHADS2 scale [9] and the CHA2DS2-VASc scale are [10] risk assessment scales for ischemic stroke in patients with atrial fibrillation, which restricts the scenarios in which they can be used. The Collaborative Research Group of the National 10th 5 Year Plan Project [11] evaluates the risk of ischemic cardiovascular diseases of Chinese using Cox proportional hazard model. Huang et al. [12] used Cox proportional hazard model combined with Hemodynamic accumulative scores to study the high-risk factors of stroke in China. Ma et al. [13] analyzed the risk factors of different gender using chi-square analysis. After the feature selection of risk factors, Weng S F et al. [14] used the machine learning classification algorithm based on the cohort data to predict the prevalance of coronary heart disease within 10 years. The results of these studies emphasize on what can be a risk factor for stroke without giving a precise definition of “high-risk”, and the methods used are too complex to operate on the huge screening population in China (especially stroke centers in rural areas). Inconvenient operations will reduce the efficiency of screening and increase the costs on training related personnel in screening.

In order to reasonably improve the coverage of rescreening, further research on the indicators of the preliminary screening is needed. The stroke screening program has accumulated a large number of screening data, laying the foundation for studying the definition of “potential high-risk”. We have used the cross-sectional stroke screening data in 2012 to conduct a preliminary study on the definition of “high-risk” based on the association of stroke and its risk factors [15]. In this paper, we will further study the causality of stroke and risk factors in middle-aged population using the cohort data during 2013 and 2017, aiming to explore whether the stroke screening and intervention program should include more indicators in the preliminary screening, such as one or two risk factors. The results of the study can directly guide stroke screening program in China, allowing staff to identify “potential high-risk” groups through questionnaires in the preliminary screening. At the same time, the method to determine “potential high-risk” can provide experience for the chronic diseases screening programs in developing countries.

## Method

### Materials

The China national stroke screening and intervention program covers Chinese residents aged 40 years and older from 31 provinces, autonomous regions, municipalities and Xinjiang Production and Construction Corps. In the screening process, a two-stage stratified cluster sampling method was adopted. Firstly, more than 200 screening areas were selected according to the local population size and total number of counties. Then, an urban community and a township are used as primary sampling units according to the geographical location and local hospital suggestions. In each primary sampling unit (PSU), all residents aged ≥40 years were surveyed during the preliminary screening period using cluster sampling [16–18]. The national stroke screening and prevention program conducts the nationwide stroke screening each year and conducts follow-up interventions on screened population every 2 years. The baseline of cohort used was set as October 2013, thus allowing all residents within the cohort to be followed-up for 5 years. Referring to the definition of the middle-aged made by WHO, we define the middle-aged as people between the ages of 40 and 59. We investigate the causal relationship of risk factors recorded in the screening in 2013 and stroke incident cases within 4 years (2014 to 2017) using the cohort. The raw data set included 73,898 middle-aged residents.

### Data cleaning

We choose middle-aged residents who never had stroke before 2014 in the cohort. We exclude stroke centers with outlier by Dixon test. The positive rate of hypertension, diabetes, atrial fibrillation and dyslipidemia is used to implement data cleaning. Firstly, we group the data by districts (east China, northeast China, north China, central China, south China, northwest China, southwest China) and urban/rural areas (14 groups in total). The Dixon test value is calculated as:

1 $$ {\mathrm{D}}_{\mathrm{low}}=\frac{{\mathrm{x}}_{\mathrm{n}}-{\mathrm{x}}_{\mathrm{n}\hbox{-} 2}}{{\mathrm{x}}_{\mathrm{n}}-{\mathrm{x}}_3}\kern0.24em \mathrm{or}\;{\mathrm{D}}_{\mathrm{high}}=\frac{{\mathrm{x}}_3-{\mathrm{x}}_1}{{\mathrm{x}}_{\mathrm{n}\hbox{-} 2}-{\mathrm{x}}_1} $$

where *x*_*1*_, *x*_*2*_, …, *x*_*n*_ (in ascending order) denotes the risk factors positive rate of each stroke centers in each group and *n* denotes the number of stroke centers in a group. Take the elimination level as *α* = 0.05 and check the table to get the critical value *D*_*1-α(n)*_. If *D*_*low*_>*D*_*1-α(n)*_, then discard outlier *x*_*1*_; if *D*_*high*_>*D*_*1-α(n)*_, then discard outlier *x*_*n*_. Repeat calculating and discarding until all values in all groups are tested.

After excluding stroke centers with outliers, we removed items with errors (i.e. error data for stroke) and items lost to follow-up (The total number of lost follow-up data is 1249, and the lost follow-up rate is 1.81%.). The analysis cohort consisted of 48,007 adults free of stroke before 2013 after cleaning. From these cases, there were 624 incident cases (1.31%) of stroke from 2014 to 2017. The first-ever stroke cases included 314 cases of cerebral infarction (about 50.3%), 57 cases of cerebral hemorrhage (about 9.1%), and 253 cases of transient ischemic attack (about 40.6%). All factors (listed in Table 1) recorded in 2013 and health status during 2013 to 2017 are inputs for the Bayesian network.

### Estimating stroke incidence

We use Bayesian network to estimate the stroke incidence (probability of stroke incident). Taking causation into consideration, the Bayesian network can better reveal the relationship between the risk factors and disease. The structure of Bayesian network is represented by directed acyclic graph, in which the nodes represents variables and the edges express the dependencies between variables. The parameter indicates the relationship between the node and its parent nodes. Parameter learning is to calculate the conditional probability distribution of each node based on collected data and given structure. Two parameter learning algorithms are commonly used: the maximum likelihood estimation algorithm and the Bayesian algorithm [19]. Due to the simplicity of the Bayesian network structure in this study, the maximum likelihood estimation algorithm [20] is chosen.

**Fig. 1:**
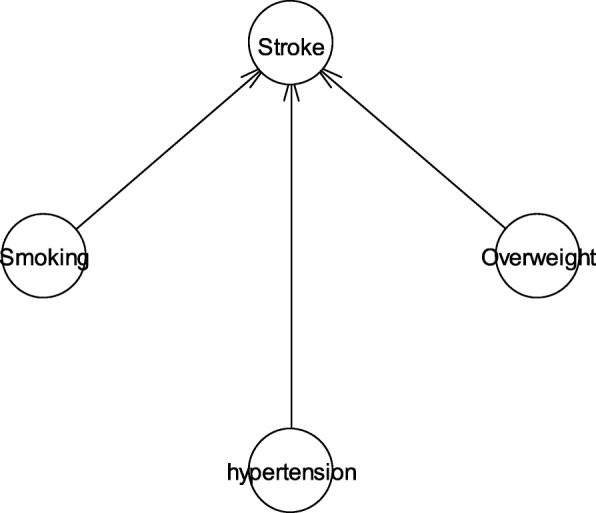
The network structure constructed with the combination of smoking, overweight and hypertension

**Fig. 2:**
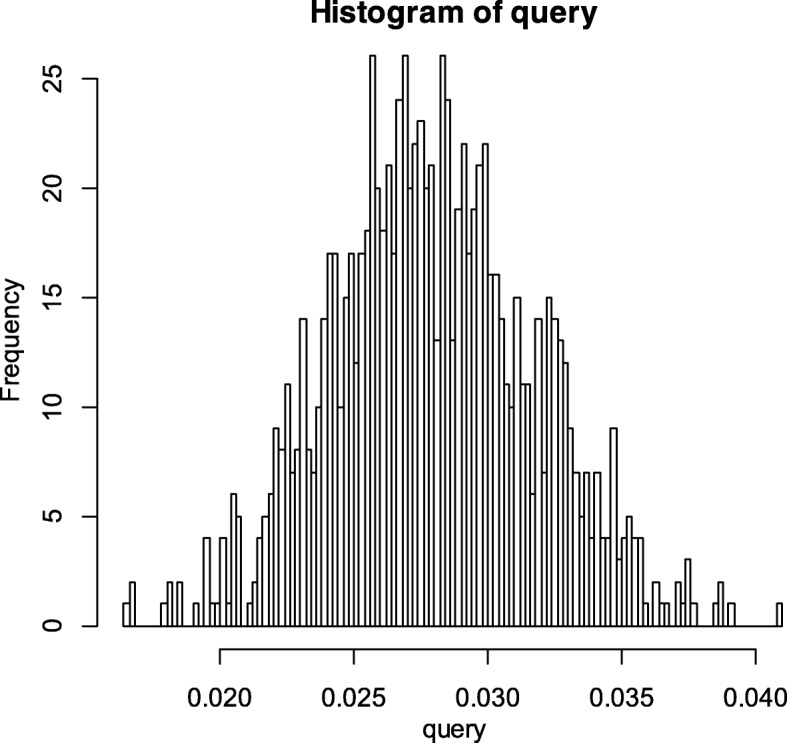
The histogram of Bayesian network inference

We study the stroke incidence in the middle-aged with one risk factor, two risk factors and three risk factors. The Bayesian network topology is constructed based on the assumption that stroke is directly caused by risk factors. Take the combination of smoking, overweight and hypertension as an example; the network structure constructed is shown in Fig. 1. We use the bnlearn [21] package in R language to learn the Bayesian network parameters and inference the stroke incidence. The inference process repeats 10,000 times, and in each iteration some of the training data is selected to calculate the probability. We use the results from the inference to further calculate the averages and 90% confidence intervals of the stroke probability. If we take hypertension as an example, the histogram of iteration results is shown in Fig. 2. The average of the inference results is 0.02138, and the 90% confidence interval is [0.02136,0.02140]. So, the first-ever stroke incidence in the middle-aged with hypertension in 4 years is about 0.02138 ± 0.00002.

## Result

The non-zero results of the inference are shown in Tables 2, 3 and 4. Among 28 calculation results with combinations of two risk factors, 21 of them are non-zero result. Among 56 calculation results with combinations of three risk factors, 31 of them are non-zero result.

**Table 2:** The first-ever stroke incidence in the middle-aged with one factor

One Factor	Probability
Hypertension	0.0214 ± 0
Atrial fibrillation	0.0206 ± 0
Smoking	0.0061 ± 0
Dyslipidemia	0.0084 ± 0
Diabetes	0.016 ± 0
Lack of exercise	0.0127 ± 0
Overweight	0.012 ± 0
Positive family history of stroke	0.0116 ± 0

**Table 3:** The first-ever stroke incidence in the middle-aged with two factors

Combinations of Factors	Probability
Dyslipidemia, Lack of exercise	0.0348 ± 0
Atrial fibrillation, Lack of exercise	0.0322 ± 0
Dyslipidemia, Diabetes	0.0307 ± 0
Overweight, Positive family history of stroke	0.0291 ± 0
Hypertension, Overweight	0.0246 ± 0
Hypertension, Dyslipidemia	0.0224 ± 0
Hypertension, Smoking	0.0202 ± 0
Smoking, Diabetes	0.02 ± 0
Hypertension, Diabetes	0.0197 ± 0
Atrial fibrillation, Overweight	0.0179 ± 0
Hypertension, Positive family history of stroke	0.0164 ± 0
Dyslipidemia, Positive family history of stroke	0.0156 ± 0
Diabetes, Overweight	0.0134 ± 0
Smoking, Overweight	0.0117 ± 0
Dyslipidemia, Overweight	0.0112 ± 0
Diabetes, Lack of exercise	0.0105 ± 0
Hypertension, Lack of exercise	0.0094 ± 0
Lack of exercise, Overweight	0.0089 ± 0
Smoking, Positive family history of stroke	0.0082 ± 0
Smoking, Lack of exercise	0.0079 ± 0
Smoking, Dyslipidemia	0.006 ± 0

**Table 4:** The first-ever stroke incidence in the middle-aged with three factors

Combinations of Factors	Probability
Atrial fibrillation, Dyslipidemia, Positive family history of stroke	0.2 ± 0.0001
Smoking, Diabetes, Positive family history of stroke	0.2 ± 0.0001
Atrial fibrillation, Dyslipidemia, Lack of exercise	0.1111 ± 0.0001
Hypertension, Atrial fibrillation, Positive family history of stroke	0.1111 ± 0.0001
Atrial fibrillation, Dyslipidemia, Overweight	0.0909 ± 0
Smoking, Diabetes, Lack of exercise	0.0909 ± 0
Smoking, Dyslipidemia, Diabetes	0.0869 ± 0
Hypertension, Diabetes, Positive family history of stroke	0.0865 ± 0
Dyslipidemia, Diabetes, Positive family history of stroke	0.0833 ± 0
Hypertension, Atrial fibrillation, Dyslipidemia	0.0741 ± 0
Hypertension, Diabetes, Overweight	0.0572 ± 0
Dyslipidemia, Overweight, Positive family history of stroke	0.0556 ± 0
Hypertension, Smoking, Dyslipidemia	0.0531 ± 0
Smoking, Dyslipidemia, Overweight	0.05 ± 0
Hypertension, Dyslipidemia, Lack of exercise	0.0459 ± 0
Dyslipidemia, Lack of exercise, Positive family history of stroke	0.0434 ± 0
Smoking, Dyslipidemia, Positive family history of stroke	0.0417 ± 0
Hypertension, Atrial fibrillation, Lack of exercise	0.04 ± 0
Dyslipidemia, Diabetes, Overweight	0.04 ± 0
Hypertension, Lack of exercise, Positive family history of stroke	0.0389 ± 0
Hypertension, Dyslipidemia, Overweight	0.0362 ± 0
Dyslipidemia, Diabetes, Lack of exercise	0.0313 ± 0
Hypertension, Lack of exercise, Overweight	0.0286 ± 0
Hypertension, Diabetes, Lack of exercise	0.0286 ± 0
Hypertension, Smoking, Overweight	0.0255 ± 0
Dyslipidemia, Lack of exercise, Overweight	0.0244 ± 0
Hypertension, Overweight, Positive family history of stroke	0.0236 ± 0
Hypertension, Dyslipidemia, Diabetes	0.0226 ± 0
Smoking, Dyslipidemia, Lack of exercise	0.0209 ± 0
Hypertension, Smoking, Positive family history of stroke	0.0179 ± 0
Hypertension, Smoking, Lack of exercise	0.0178 ± 0

The results in Table 2 show that the three most important risk factors are hypertension, atrial fibrillation and diabetes, which is consistent with previous research results. High blood pressure has long been regarded as a major risk factor for stroke [22]. He J et al. [23] found that the overall relative risk of stroke associated with hypertension was 5.43 from a meta-analysis. A national study in China shows hypertension remains the most important risk factor for all types of strokes [2]. Based on cohort of Chinese atrial fibrillation patients in Hong Kong with detailed long-term follow-up, Chung-Wah Siu et al. [24] concluded that Chinese atrial fibrillation patients are at high risk for ischemic stroke. Chern-En et al. [25] studied data of atrial fibrillation patients in Asia and found that they had higher stroke rate. Fiona Bragg et al. [26] recruited 0.5 million Chinese adults and found that individuals with diabetes (previously or newly diagnosed) had 1.5- to 2.5-fold higher risks of developing ischemic heart disease and stroke (both ischemic and hemorrhagic). Previous studied in western countries have made similar conclusions [27, 28].

## Discussion

We calculated that the crude incidence of first-ever stroke in Chinese middle-aged adults without any of the risk factors mentioned above within 4 years is about 0.0070. On the whole, the incidence with three risk factors is significantly higher than that with two risk factors, and the incidence with two risk factors is significantly higher than that with only one risk factor. For example, the first highest incidence with three risk factors (the incidence with atrial fibrillation, dyslipidemia, and positive family history of stroke is 0.2) is 5.76 times higher than the first highest incidence with two risk factors (the incidence with dyslipidemia and lack of exercise is 0.0348) and 28.6 times higher than the incidence without any risk factor. The median of incidence with three risk factors (the incidence with hypertension, dyslipidemia, diabetes is 0.0226) is 1.9 times higher than the median of incidence with two risk factors (the incidence with smoking, overweight is 0.0117) and 3 times higher than the incidence without any risk factor, and it is also higher than the sixth-highest of the incidences with two risk factors. Ten of the incidences with three risk factors (the 10th incidence, the incidence with hypertension, atrial fibrillation and dyslipidemia, is 0.0741) are 10 times higher than the incidence without any risk factor, and those with these factor combinations should be paid focal attention in stroke prevention.

On the other hand, it can be concluded that some stroke incidences with certain combinations of two risk factors can be higher than that with combinations of three risk factors. For example, the first-ever stroke incidence for the middle-aged with diabetes and dyslipidemia is 0.03074, while the incidence with smoking, dyslipidemia, lack of exercise is 0.0209. Table 3 shows that the incidences with dyslipidemia and lack of exercise, atrial fibrillation and lack of exercise, dyslipidemia and diabetes, overweight and positive family history of stroke, hypertension and overweight, hypertension and dyslipidemia are 3 times higher than the incidence without any risk factor, and the first four of the incidences are 4 times higher than the incidence without any risk factor. The incidence with a single risk factor can be higher than that with the combinations of three risk factors in some cases. For example, the first-ever stroke incidence with hypertension is 0.0214(3 times higher than the incidence without any risk factor). Correspondingly, we obtained similar results that stroke probability with one factor or a combination of two risk factors can be higher than that with a combination of three risk factors in the previous study [15].

Therefore, for middle-aged people, the indicators used in the screening are not reasonable enough. Some new criteria with one important factor or combinations of two factors should be added so as to avoid missing middle-aged people with high risk of stroke.

People with chronic disease are more likely to develop stroke. AleksandraZeljkovic et al. [29] found that acute ischemic stroke was associated with adverse distributions of low-density lipoprotein and small-sized high-density lipoprotein subclasses. In addition, short-term mortality after acute ischemic stroke was associated with increased small, dense low-density lipoprotein particles. Besides, unhealthy lifestyle is also a risk factor for stroke [2, 30–32]. The screening program staff should also provide people with risk factors advices to change their unhealthy lifestyle since lifestyle intervention may lower their incidence of stroke [33].

Taking these existing research results into account, we can take the ratio of the stroke incidence with combinations of risk factors and incidence without any of the risk factors as a variable threshold. If we take the threshold as 2.5(stroke incidence with hypertension, smoking, lack of exercise in the last row of Table 4 is 0.0178, which is about 2.5 times the crude incidence without risk factors), we should consider to add four more two-factor combinations (hypertension and smoking, smoking and diabetes, hypertension and diabetes, atrial fibrillation and overweight) and two more single risk factors (hypertension, atrial fibrillation). Combining with other results of epidemiological studies, researchers can adjust the threshold to achieve a balance between economy and efficiency.

The results show that the stroke incidences with a small number of combinations of two or three risk factors are quite low (even zero). Since patients with certain two or three risk factors are infrequent in the population, there may be bias in the results of the stroke incidence with these risk factors, which will be studied in further research. At the same time, we conducted health education on the population considered at medium or high risk of stroke since the first screening in 2013 (such as smoking cessation and low-salt diets), so the results of stroke incidence of high-risk groups may theoretically be lower than the cohort without intervention. Even so, the incidence with the risk factors we discovered is 2.5 times higher than the incidence without any risk factor. The stroke incidence of the population considered at medium or high risk in first screening would have been higher without the intervention. Therefore, the threshold of 2.5 we take is a conservative estimate.

## Conclusion

In this paper, we use Bayesian network to study first-ever stroke incidence with risk factors for the middle-aged and explore the rationality of the classification criteria used in the preliminary screening proposed by stroke screening and intervention program. The results show that it is not reasonable to simply group the middle-aged with three risk factors to “potential high-risk” catalog in the preliminary screening. Combinations with some chronic diseases can significantly increase the probability of stroke. There is a need for national stroke screening and intervention program to further consider modifying the screening criteria in the preliminary screening to include new criteria with one important factor or combinations of two factors.
